# Inactivation of respiratory syncytial virus by zinc finger reactive compounds

**DOI:** 10.1186/1743-422X-7-20

**Published:** 2010-01-26

**Authors:** Marina S Boukhvalova, Gregory A Prince, Jorge CG Blanco

**Affiliations:** 1 Virion Systems, Inc. 9610 Medical Center Drive, Suite 100, Rockville, MD, USA

## Abstract

**Background:**

Infectivity of retroviruses such as HIV-1 and MuLV can be abrogated by compounds targeting zinc finger motif in viral nucleocapsid protein (NC), involved in controlling the processivity of reverse transcription and virus infectivity. Although a member of a different viral family (*Pneumoviridae*), respiratory syncytial virus (RSV) contains a zinc finger protein M2-1 also involved in control of viral polymerase processivity. Given the functional similarity between the two proteins, it was possible that zinc finger-reactive compounds inactivating retroviruses would have a similar effect against RSV by targeting RSV M2-1 protein. Moreover, inactivation of RSV through modification of an internal protein could yield a safer whole virus vaccine than that produced by RSV inactivation with formalin which modifies surface proteins.

**Results:**

Three compounds were evaluated for their ability to reduce RSV infectivity: 2,2'-dithiodipyridine (AT-2), tetraethylthiuram disulfide and tetramethylthiuram disulfide. All three were capable of inactivating RSV, with AT-2 being the most potent. The mechanism of action of AT-2 was analyzed and it was found that AT-2 treatment indeed results in the modification of RSV M2-1. Altered intramolecular disulfide bond formation in M2-1 protein of AT-2-treated RSV virions might have been responsible for abrogation of RSV infectivity. AT-2-inactivated RSV was found to be moderately immunogenic in the cotton rats *S.hispidus *and did not cause a vaccine-enhancement seen in animals vaccinated with formalin-inactivated RSV. Increasing immunogenicity of AT-2-inactivated RSV by adjuvant (Ribi), however, led to vaccine-enhanced disease.

**Conclusions:**

This work presents evidence that compounds that inactivate retroviruses by targeting the zinc finger motif in their nucleocapsid proteins are also effective against RSV. AT-2-inactivated RSV vaccine is not strongly immunogenic in the absence of adjuvants. In the adjuvanted form, however, vaccine induces immunopathologic response. The mere preservation of surface antigens of RSV, therefore may not be sufficient to produce a highly-efficacious inactivated virus vaccine that does not lead to an atypical disease.

## Background

Vaccines for numerous infectious diseases have been developed using whole inactivated virions. Some of the successful examples include inactivated hepatitis A vaccine [1], poliovirus vaccine [2], and SIV vaccine [3]. While providing effective protection in some cases, inactivated virus vaccines are sometimes associated with the exacerbation of the disease. For example, formalin-inactivated respiratory syncytial virus (RSV) vaccine administered to infants and children in the United States in the 1960's resulted in the enhancement of the disease after subsequent exposure of these children to RSV [4]. Similarly, formalin-inactivated measles vaccine, also developed in the 1960's, caused a severe and atypical form of measles following exposure to the wild type measles virus [5]. While the exact cause of such atypical responses to formalin-inactivated viruses is not known, one possible explanation is that traditional means of viral inactivation, such as formalin or heat treatment can denature virion surface proteins [6]. This would result in altered antigenicity of a virus, accompanied by an atypical and often harmful host response to infection. Viral inactivation with maximal preservation of its original structure may thus provide a solution for successful vaccine development against diseases currently refractory to vaccination.

In the past, one particularly interesting method for such "preserving" viral inactivation has been developed. The target of this method have been nucleocapsid (NC) proteins of retroviruses, such as a human immunodeficiency virus 1 (HIV-1), murine leukemia virus (MuLV) and simian immunodeficiency virus (SIV). The nucleocapsid proteins of these viruses are small basic proteins that bind single-stranded nucleic acids and increase synthesis of full length DNA during the reverse transcription reaction. All retroviral NC proteins (with an exception of spumaretrovirus group [7]) contain one or two copies of a zinc finger motif. This zinc finger motif is essential for viral replication, as mutations in the zinc-coordinating residues lead to the loss of infectivity and significant reduction of genomic RNA packaging [8-10]. The exact mechanism by which the NC protein operates is not known, but it is thought that the NC protein acts as a nucleic acid chaperone that facilitates initiation of reverse transcription and serves to reduce pausing by the reverse transcriptase to ensure efficient synthesis of full-length DNA during virus replication [11,12]. Moreover, residues within and flanking the zinc finger in the NC protein are responsible for processivity of reverse transcription reaction [12]. The conserved nature of the zinc finger motifs, as well as its crucial role in a viral life cycle, made NC proteins an attractive target for development of antiretroviral drugs. In particular, several compounds have been identified that covalently modify the NC zinc fingers resulting in ejection of Zn^2+ ^and loss of infectivity [13-15]. Detailed analysis of HIV-1 inactivation by one of such compounds, namely aldrithiol-2 (AT-2) has been studied in considerable detail and revealed that while the viral infectivity was abrogated, virions were able to enter the target cells and the virion surface proteins retained structural and functional integrity [16].

A number of reasons prompted us to believe that a similar approach for virus inactivation can be applied to the respiratory syncytial virus (RSV). RSV is a member of the genus *Pneumovirus *that contains a negative-strand RNA genome encoding 11 proteins. One of these proteins, M2-1, contains a zinc finger motif. This protein apparently serves the same role in RSV as the nucleocapsid protein does in retroviruses: it controls processivity of viral polymerase. In particular, M2-1 prevents premature termination during transcription of the viral mRNAs [17,18]. M2-1 is a 22 kDa protein encoded by the first (upstream) ORF of M2 mRNA. The zinc finger motif is located in the N-terminus of the protein, from residues 7 to 25. Just as retroviral NC proteins are important for retroviruses, M2-1 is required for RSV to produce infectious particles [19]. Moreover, the intact zinc finger motif of the M2-1 protein is required for maintaining functional integrity of the protein [20]. The zinc finger motifs of RSV M2-1 protein and NC proteins of retroviruses have slightly different consensus sequences: C-X_7_-C-X_5_-C-X_3_-H (so-called Cys_3_-His_1 _motif) for M2-1 protein and C-X_2_-C-X_4_-H-X_4_-C (CCHC motif) for NC proteins, where X denotes variable amino acids [21,22]. The Cys_3_-His_1 _motif is found in M2-1 proteins of all the pneumoviruses examined up to date [20]. This motif is also present in the human zinc-binding protein, Nup475, involved in regulation of mRNA stability [23,24]. The structure of the Nup475 Cys_3_-His_1 _motif has been proposed based on nuclear magnetic resonance and photometric analysis, revealing that the Zn^2+ ^ion is coordinated by three conserved cystein residues and one histidine residue in the Cys_3_-His_1 _motif [23,25]. Although the sequence of the zinc finger CCHC motif of NC proteins differs from that of the Cys_3_-His_1 _motif, Zn^2+ ^ions bind to the CCHC motifs also through coordination with the three cystein and one histidine residues [21,22]. Thus, in spite of the difference in the length of the loops connecting Zn-coordinating residues, Zn^2+ ^ions appear to bind in a similar manner to both Cys_3_-His_1 _and CCHC motifs.

Given the functional similarity between the zinc finger containing proteins of retroviruses and RSV, and similarity in basic architecture of the zinc finger motifs in these proteins, we have noted a possibility that some of the chemicals identified as reactive towards the zinc finger motif in the NC proteins might also react with the zinc finger motif of the M2-1 protein of RSV. Therefore, we have tested some of the zinc finger reactive compounds that were discovered as active against HIV-1 and MuLV in the experiments using respiratory syncytial virus. This paper reports that the zinc finger reactive compounds capable of inactivating HIV-1 and other retroviruses by targeting their nucleocapsid protein are also capable of inhibiting RSV infectivity, and that this inhibition is accompanied by modification of the RSV zinc finger containing protein M2-1. This type of inactivation might allow maximum preservation of the virion surface structure, a feature important in view of future vaccine development.

## Results

### Zinc-finger-reactive compounds are effective against RSV as measured by their effect on infectivity

**Figure 1:**
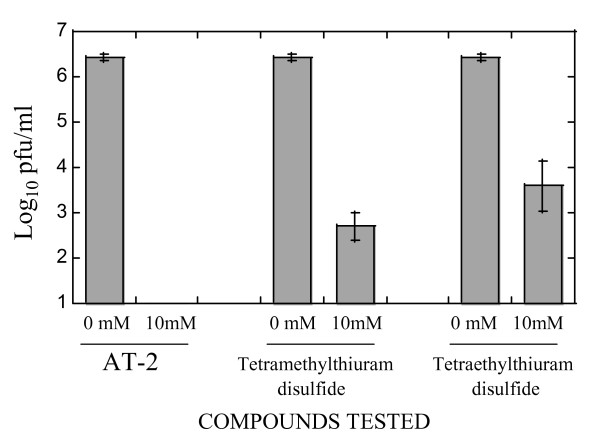
**Inactivation of respiratory syncytial virus by AT-2, Tetramethylthiuram disulfide, and Tetraethylthiuram disulfide**. RSV was treated with 10 mM of the indicated compounds for 24 hrs at 37°C. Viral titers were then determined by plaque assay on HEp-2 cells. Control reactions (corresponding to "0 mM" of each chemical) contained the same amount of DMSO as that present in drug-containing incubations.

Three compounds were chosen for evaluation of their effect on RSV infectivity: 2,2'-dithiodipyridine (aldrithiol-2; hereafter referred to as AT-2), tetraethylthiuram disulfide and tetramethylthiuram disulfide. These chemicals were previously demonstrated to inactivate HIV-1 and MuLV through targeting their nucleocapsid zinc finger proteins [13,15,16]. The effect of AT-2, tetraethylthiuram disulfide and tetramethylthiuram disulfide on the infectivity of RSV was tested by incubating RSV with each one of these chemicals and then assessing remaining RSV infectivity on HEp-2 cells. All three chemicals effectively reduced RSV infectivity, with AT-2 being the most potent inhibitor (Figure 1). At the concentration of 10 mM, AT-2 decreases RSV infectivity to below the detection level (10 pfu/ml). Tetraethylthiuram disulfide and tetramethylthiuram disulfide, while significantly reducing infectivity of RSV, were not as effective as AT-2 at similar concentration. The effect of higher concentrations of these two chemicals on RSV infectivity, however, was impossible to test due to their limited solubility (data not shown).

**Figure 2:**
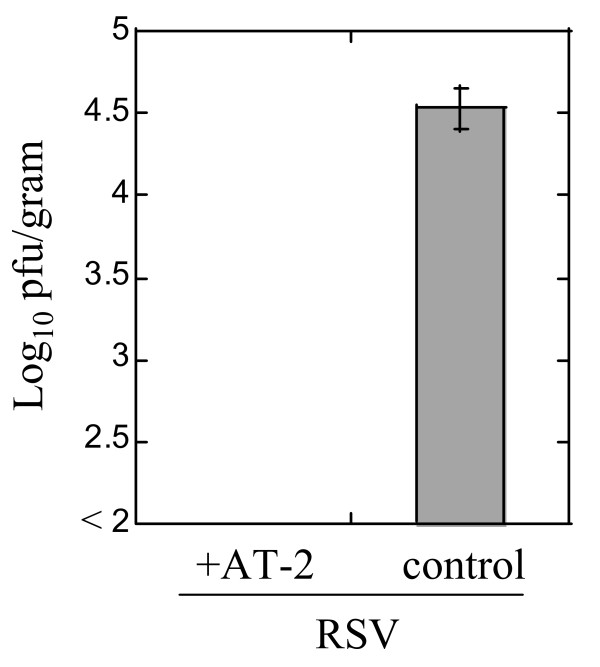
**Inability of respiratory syncytial virus inactivated with AT-2 to replicate in the lungs of cotton rats**. RSV was inactivated by incubation with 10 mM AT-2 for 24 hrs at 37°C. Virus was then diluted with PBS and used to intranasally infect cotton rats. Control animals received sham-treated RSV. Four days post-challenge animals were sacrificed and lungs were collected for viral titrations. Results shown are the mean log (viral titer) ± SE for 4 animals per group.

Infectivity of AT-2-inactivated RSV was also assessed *in vivo *using cotton rats (Figure 2). Animals were intranasally inoculated with RSV inactivated by 10 mM AT-2 for 24 hrs, or sham-treated for 24 hrs. Four days later, animals were sacrificed and viral replication in the lungs evaluated. No infectious viral particles were detected in the lungs of animals inoculated with AT-2-inactivated RSV, while sham-treated RSV replicated efficiently in the lungs of infected animals (Figure 2).

### Dynamics of RSV inactivation by AT-2

**Figure 3:**
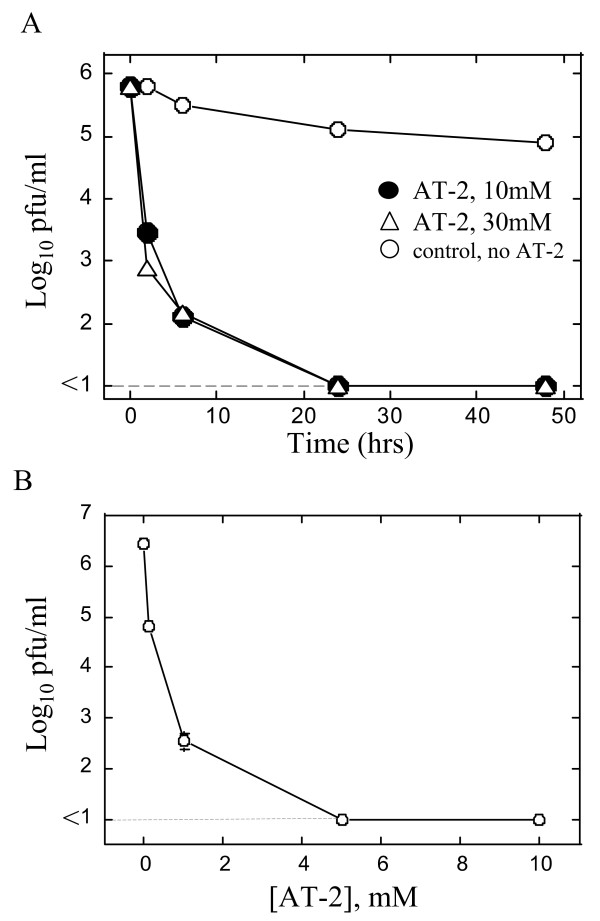
**Time- and dose-dependence of RSV inactivation by AT-2**. (A) Time course of RSV inactivation. RSV was incubated with 10 mM, 30 mM AT-2 or the corresponding amount of DMSO (control, no AT-2) at 37°C for different times. At the indicated time points, reaction was stopped and viral infectivity was determined by plaque assay on HEp-2 cells. Dashed line indicates the limit of the sensitivity of titration assay. (B) Dose response curve of RSV inactivation by AT-2. RSV was incubated with the indicated amounts of AT-2 at 37°C for 24 hrs. Viral titers were determined by plaque assay on HEp-2 cells. Dashed line indicates the limit of the sensitivity of titration assay.

Effect of AT-2 on RSV infectivity was investigated in more detail following the initial observation that AT-2 was the compound most effective at inhibiting RSV (Figure 3). First, the time-dependency of RSV inactivation by AT-2 was explored. Inactivation reactions containing 10 mM or 30 mM AT-2 were set up and allowed to proceed for 2, 6, 24 or 48 hours. In the absence of AT-2 treatment, sucrose-stabilized RSV was rather stable (Figure 3A), consistent with earlier observations using a similarly prepared and stabilized RSV [26]. Incubation of this virus for as long as 48 hrs at 37°C diminished RSV infectivity by less than one Log_10 _pfu/ml. When RSV was incubated in the presence of AT-2, however, inactivation of virus was visible within the first 2 hours of incubation. In that time period, RSV infectivity diminished by 2 or 3 Log_10 _pfu/ml when 10 mM and 30 mM AT-2 were used, respectively. 24 hour incubation with AT-2 resulted in no detectable infectious viral particles left. Inactivation of RSV by AT-2 did not appear to follow single-hit kinetics, in contrast to RSV inactivation by irradiation with ultraviolet light [27]. 30 mM AT-2, while producing higher inactivation of RSV at 2 hrs, did not significantly increase RSV inactivation compared to the lower, 10 mM AT-2 concentration, probably due to the fact that solubility of AT-2 at 30 mM was already limited (data not shown). Twenty four hour inactivation period, therefore, was identified as the one sufficient for complete inactivation of RSV by concentrations of AT-2 equal to or higher than 10 mM. The dose-dependence of inactivation was determined next by incubating RSV with various amounts of AT-2 for 24 hrs (Figure 3B). AT-2 in the concentration of 5 mM or higher diminished RSV infectivity to below the detection level. At the lowest dose tested, 100 nM AT-2, RSV infectivity was reduced by over 1.5 Log_10 _pfu/ml.

### AT-2 treatment of RSV results in the modification of the RSV M2-1 protein

**Figure 4:**
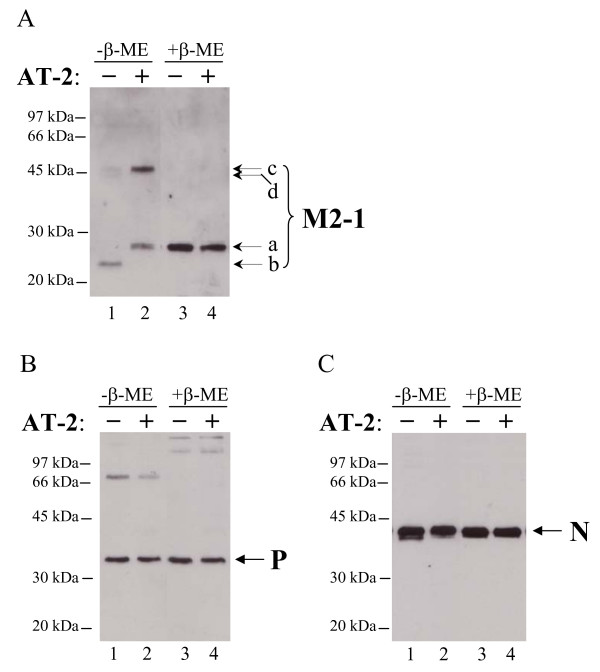
**Respiratory syncytial virus inactivation by AT-2 is accompanied by modification of the M2-1 protein**. RSV was inactivated by incubation with 10 mM AT-2 for 24 hrs at 37°C (lanes 2 and 4), or with corresponding amount of DMSO (lanes 1 and 3). At the end of the incubation, RSV particles were lysed and analyzed by SDS-PAGE either in the presence (lanes 3 and 4), or in the absence (lanes 1 and 2) of β-ME, followed by immunoblotting with IgY against RSV: (A) M2-1 protein, (B) P protein, and (C) N protein.

Several research groups have demonstrated that zinc finger reactive compounds that inactivate retroviruses do so by targeting their zinc finger motif containing nucleocapsid proteins. These compounds were shown to penetrate the viral envelope of cell-free HIV-1 and MuLV and react with nucleocapsid protein of these viruses [14,15,28], causing formation of intra- and intermolecular disulfide crosslinks. As M2-1 is the only protein of RSV containing a zinc finger motif, and the most likely target of the AT-2 action, we set to determine whether a similar modification of the M2-1 protein occurs following AT-2 treatment of cell-free RSV. To address that question, AT-2 treated RSV particles were lysed and fractionated by sodium dodecyl sulfate-polyacrylamide gel electrophoresis (SDS-PAGE) either in the presence or in the absence of β-ME. The proteins were then analyzed by Western blot using IgY antibodies against RSV antigens (Figure 4).

M2-1 protein in RSV-infected cells has been reported to migrate as multiple species. This variability is due in part to the formation of intramolecular disulfide bonds [29], as well as to the existence of the phosphorylated form of M2-1 protein [20]. Less is known about electrophoretic behavior of M2-1 protein present in cell-free RSV virions. In the presence of β-ME we detected a single band corresponding to M2-1 protein in untreated RSV virions, designated as the band "a" in Figure 4A (lane 3). This band migrates with an apparent molecular weight of ~26-27 kDa and corresponds in size to the main M2-1 band detected with the same antibody in RSV-infected HEp-2 and A549 cells (data not shown). In the absence of β-ME, however, several M2-1 bands are visible in cell-free RSV (bands "b", "d", and "c" in Figure 4A, lane 1). Band "b" contained the majority of M2-1 molecules and based on its apparent molecular weight corresponds to the monomeric form of M2-1 protein. Bands "c" and "d", although barely visible, most likely represent a dimer of M2-1 stabilized by intermolecular disulfide crosslinks, as these bands disappeared in the presence of β-ME (compare lane 1 to lane 3). M2-1 protein of RSV in the absence of β-ME (band "b", lane 1) has a slightly better electrophoretic mobility than in the presence of β-ME (band "a", lane 2), suggesting that it might be a conformational form stabilized by intramolecular disulfide bonds.

In the presence of β-ME, M2-1 protein of virions treated with AT-2 had the same electrophoretic mobility as M2-1 protein of untreated, control virus (Figure 4A, compare lane 4 to lane 3). In the absence of β-ME, however, electrophorectic mobility of M2-1 was significantly different. AT-2 treatment had converted a substantial fraction of the M2-1 protein into a higher-molecular-weight band of approximately 46 kDa, evidently a dimer of M2-1 protein (band "c", lane 2). The disappearance of this band following β-ME treatment also suggests that it might be disulfide-bond crosslinked dimer of M2-1. Moreover, a band corresponding to a monomer of M2-1 protein in untreated RSV virions (band "b") had shifted to a slightly higher position following AT-2 treatment, acquiring electrophoretic mobility similar to that of reduced M2-1 protein species in both untreated RSV and in virions treated with AT-2 (band "a"). Assuming that the band "b" in untreated RSV virions represents a M2-1 form stabilized by intramolecular disulfide bonds, the disappearance of this band following AT-2 treatment reflects modification of cystein residues previously involved in formation of these disulfide bonds.

To determine whether AT-2 treatment in addition to modifying M2-1 protein might have caused a modification of other RSV proteins, we have used chicken IgY antibodies to several RSV proteins in immunoblots of fractionated treated/untreated virions. Figures 4B and 4C show representative immunoblots of RSV P and N proteins, respectively. It can be seen that neither P, nor N protein appear to be modified by AT-2 treatment of RSV, as evidenced by similar electrophoretic behavior of these proteins both in AT-2-treated and in control preparation under reducing or non-reducing conditions. Similarly, no modification of RSV F or G proteins was detected following AT-2 treatment (data not shown). Moreover, treatment of RSV with AT-2 has not apparently affected the total amount of virus following inactivation, as comparable levels of structural RSV proteins were detected in both AT-2-treated and control, untreated RSV preparations.

### Immunogenicity of AT-2-inactivated RSV

**Figure 5:**
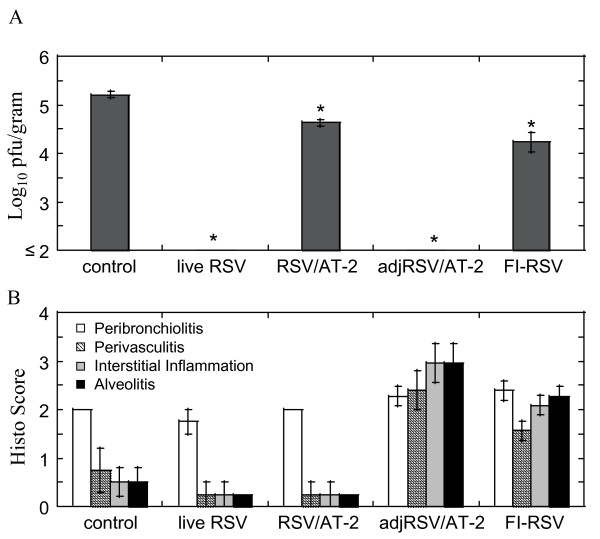
**Immunogenicity of AT-2-inactivated RSV in the cotton rat model**. Cotton rats were immunized intramuscularly with AT-2-inactivated RSV ("RSV/AT-2"), AT-2-inactivated RSV adjuvanted with Ribi emulsion (adjRSV/AT-2) or formalin-inactivated RSV (FI-RSV). Control animals remained unvaccinated ("control") or were immunized via repeated infection with live RSV ("live RSV"). All animals were challenged with RSV and sacrificed 4 days after infection for analysis of pulmonary viral load (A) and histopathology (B). Results are the mean ± SEM for 4 animals per group. *p < 0.05 when compared to the "control" group.

Modification of surface proteins during inactivation of RSV by formalin is thought to contribute to the development of an atypical enhanced disease during subsequent RSV infection [6]. AT-2 targets an internal viral protein while preserving surface proteins and may therefore generate a safer RSV vaccine. To test this hypothesis, we have addressed efficacy and safety of AT-2-inactivated RSV in the cotton rat *S.hispidus *model (Figure 5). Immunogenicity of AT-2-inactivated RSV (RSV/AT-2) was compared to that of formalin-inactivated RSV (FI-RSV) and to immunity induced by repeat live RSV infection (live RSV). When administered in the absence of any adjuvants, AT-2 inactivated RSV conferred a moderate but statistically-significant reduction of RSV load in the lung (Figure 5A). The extend of this reduction was comparable to that caused by FI-RSV, however no histopathological response associated with formalin-inactivated virus was seen with AT-2-inactivated RSV (Figure 5B). To improve immunogenicity of AT-2-inactivated RSV, Ribi adjuvant was included into vaccine formulation. The resulting vaccine (adjRSV/AT-2) afforded complete protection of the lung from RSV replication (Figure 5A), but also caused a vaccine-enhanced disease (Figure 5B).

## Discussion

Human Respiratory Syncytial Virus is a member of the *Pneumovirus *genus of the family *Paramyxoviridae*. Replication of paramyxoviruses is a complex process that relies on transcription of virally-encoded proteins tightly linked to replication of viral genome through the synthesis of antigenome intermediate. The RNA polymerase complex involved in transcription and replication of RSV genome consists of the N, P and L proteins [30,31], but requires an additional protein, M2-1, to ensure efficient transcription and replication.

M2-1 is a 194 amino acid protein required for synthesis of RSV RNA. This protein is an antitermination factor that increases processivity of viral RNA polymerase at gene junctions and prevents premature termination during transcription [17,18,20,32]. M2-1 protein in RSV-infected cells can exist in a phosphorylated form, and is capable of binding to the nucleocapsid protein of RSV [20]. The importance of both of these features for the function of M2-1 protein is currently unknown. The N-terminal part of the M2-1 protein contains a Cys_3_-His_1 _motif (C-X_7_-C-X_5_-C-X_3_-H). This motif is present in all currently known pneumoviruses [33]. A similar motif is present in mammalian transcription factor Nup475, where it was shown to bind zinc [23]. The Cys_3_-His_1 _motif of M2-1 protein is required for its function, as mutations at some of the predicted zinc-coordinating residues prevent M2-1 from enhancing transcriptional read-through, alter M2-1 phosphorylation state and prevent M2-1 interaction with the nucleocapsid protein in transfected cells [20,34].

The Cys_3_-His_1 _motif of the RSV M2-1 protein differs in consensus sequence from the conserved CCHC zinc finger motif of retroviral nucleocapsid proteins. Yet, the M2-1 protein of human respiratory syncytial virus displays functional similarity to the nucleocapsid protein of retroviruses such as HIV-1 and MuLV: both proteins control processivity of viral polymerase, both are required to produce infectious viral particles, and both are functionally dependent on intact zinc finger motif. This paper presents evidence that compounds that inactivate retroviruses by targeting the zinc finger motif in their nucleocapsid proteins are also effective against human respiratory syncytial virus. A detailed analysis of the effect of one of such compounds, namely, AT-2 on RSV infectivity is described, and it reveals that RSV inactivation by AT-2 in accompanied by modification of the M2-1 protein. The fact that a compound acting by covalently modifying zinc finger motif containing proteins of retroviruses can inactivate a pneumovirus through targeting a zinc finger motif containing protein of seemingly analogous function, suggests that mechanisms of viral transcription and replication might be more conserved than currently appreciated, and might point to an evolutionary link between viruses of genus *Pneumovirus *and some retroviruses. Significant inactivation of RSV by AT-2, however, required extending the inactivation time to as long as 24 hours. While such a prolonged incubation does not dramatically affect infectivity of control sucrose-stabilized RSV preparation, it might not be an acceptable approach for un-stabilized RSV that is heat-labile [26]. Chemicals other than AT-2, but with similar mode of action might be more effective in inactivating RSV faster. Zinc finger reactive compounds inhibiting HIV-1 infectivity have been identified by the National Cancer Institute's drug screening program [14]. A similar type of approach might be needed to identify compounds that target more efficiently the zinc finger motif of RSV and other pneumoviruses.

Numerous methods of inactivation of respiratory syncytial virus have been attempted in the quest for a safe and efficient RSV vaccine. Each one of them, however, has been plagued by its own limitations. Chemical methods of RSV inactivation, such as formalin treatment, have led to unexpected enhancement of pulmonary disease in vaccines that have acquired natural RSV infection following vaccination [4,5]. Subunit RSV vaccines often lack sufficient antigenicity, while development of attenuated replicating RSV vaccines has been complicated by their residual virulence and genetic instability [35-37]. The method of chemical inactivation of respiratory syncytial virus described here carries at least two advantages when compared to other methods of RSV inactivation. First, a large scale production of RSV inactivated by means of chemical targeting its M2-1 protein is easily attainable, as a viral pool of replication-competent virus is grown prior to its inactivation. This contrasts with the complication associated with the live attenuated RSV vaccine candidates, which are often less efficient in replication. Second, a chemical used for inactivation (AT-2 in this case) penetrates the viral envelope and targets a viral protein that is located inside the viral particle, rather than proteins located on its surface. This type of inactivation might spare surface-exposed molecules often serving as antigenicity determinants. In fact, the harmful effect of formalin-inactivated RSV vaccine is believed to be associated with the adverse modification of surface RSV proteins following formalin treatment [6]. Our results show that AT-2-inactivated RSV is immunogenic and that at the doses affording protection comparable to protection induced by FI-RSV vaccine it does not cause a vaccine-enhanced disease. These results support the hypothesis that modification of surface antigens by formalin may be contributing to the development of an atypical disease [6]. The potential contribution of alum contained in FI-RSV but not RSV/AT-2 vaccine to a development of vaccine-enhanced disease, however, cannot be ruled out [38]. This work also demonstrates that AT-2-inactivated RSV is only moderately immunogenic and that inclusion of an adjuvant is needed to improve its efficacy. This is not surprising as most subunit and inactivated RSV vaccines generated so far and administered parenterally do not induce a robust immune response in the absence of an adjuvant [39]. Increasing immunogenicity of AT-2-inactivated RSV also increases the potential of this vaccine to cause vaccine-enhanced disease, suggesting that preservation of surface antigenic determinates of inactivated RSV is not sufficient to create a highly efficacious and safe whole virus vaccine against RSV disease.

Respiratory syncytial virus inactivated by an approach described in this paper might present a valuable tool for investigation into the mechanisms of RSV action. Pathogenesis of respiratory syncytial virus infection depends in large part on host immune and inflammatory response to the virus. Events triggered by the initial contact of the virus with cells of the innate immune system might ultimately be responsible for the outcome of the infection. Targeted inactivation of RSV with possible preservation of the majority of structural proteins, combined with the ease of production of such inactivated viral particles, might help to advance our understanding of RSV biology.

## Conclusions

This work demonstrates that compounds inactivating retroviruses through targeting their zinc finger-containing NC proteins can also inactivate RSV. Inactivation of RSV by AT-2 is accompanied by a significant modification of RSV M2-1, a change that is likely contributing to the loss of viral infectivity. Inactivated virus in the absence of adjuvants is moderately immunogenic and does not cause a vaccine-enhanced disease in contrast to formalin-inactivated RSV. Addition of Ribi adjuvant dramatically increases immunogenicity of AT-2-inactivated RSV, but also leads to enhanced pulmonary pathology, limiting the vaccine potential of RSV inactivated with zinc-finger-reactive compounds. AT-2 inactivated RS, however, may present an important research tool for understanding immunobiology of RSV disease.

## Methods

### Viruses and Cells

Long strain of RSV was obtained from American Type Culture Collection, Manassas, VA and propagated in HEp-2 cells after serial plaque purification. A pool of virus containing 10^7.5 ^pfu/ml in stabilizing media composed of 25% sucrose and 2% FBS in PBS, pH 7.4 [26] was used for all experiments. Viral titers were determined by plaque assay using HEp-2 cells as described in Prince et al., 1978 [40].

### Chemicals and Antibodies

Aldrithiol-2 (AT-2), Tetraethylthiuram disulfide and Tetramethylthiuram disulfide were purchased from Sigma-Aldrich. AT-2 was reconstituted in 100% DMSO to a stock concentration of 300 mM. Tetraethylthiuram disulfide and tetramethylthiuram disulfide were reconstituted at 169 mM and 208 mM, respectively, using DMSO as a solvent as well. Unless otherwise stated, further dilutions of chemicals were done in PBS, pH 7.4. Chicken IgY antibodies against various RSV proteins were kindly provided by Robert Brazas (currently at Epigenetics).

### Vaccines and Adjuvants

FI-RSV vaccine Lot 100 was kindly provided by Dr. Hyun-Wha Kim, one of the participants in the clinical trials in 1965-1967 [4]. FI-RSV preparation contained alum and has been maintained under refrigeration since the time of its manufacture. Ribi adjuvant (MPL, TDM, CWS Emulsion) was purchased from Sigma-Aldrich.

### Chemical RSV inactivation

Frozen virus stocks were thawed at 37°C in a water bath. Inactivation reactions were carried in 200 μl total volume, using PBS, pH 7.4 as a buffer. Each reaction contained 20 μl of 10^7.5 ^pfu/ml RSV Long, and appropriate amount of chemical stock to yield the working concentration of 1-30 mM. Control reactions contained amount of DMSO equal to that present in the drug-containing incubations (up to 10% DMSO). Reactions were incubated in 37°C air-incubator with constant shaking at 225 rpm for 2-24 hrs and vortexed every 2 hrs during the first 8 hrs to disaggregate the virus. At the end of the incubation period, virus was concentrated and chemicals were removed by ultrafiltration against PBS, pH 7.4 using Centricon YM-100 columns (Millipore). Samples were then snap-frozen on dry ice and stored at -70°C.

### Animal experiments

Adult inbred cotton rats (*S. hispidus*) were obtained from the colony maintained at Virion Systems, Inc. (Rockville, MD). Animals were fed a standard diet of rodent chow and water and housed in large polycarbonate cages. For the assessment of AT-2-inactivated-RSV ability for pulmonary replication, two groups of animals (four animals each) were used. RSV was inactivated by incubation with 10 mM AT-2 for 24 hrs at 37°C as outlined above. Control virus preparation was incubated in parallel with AT-2-containing reaction, but in the absence of AT-2. Animals were inoculated intranasally under isoflurane anesthesia with 100 μl/animal of sham-treated RSV or with 100 μl/animal of the AT-2-inactivated RSV, both originally containing 10^5 ^pfu RSV Long. On day 4 post-challenge animals were killed by carbon dioxide intoxication. Lungs were extracted and homogenized for virus quantification.

Immunogenicity of AT-2-inactivated RSV was tested via parenteral route. Groups of 4-5 *S.hispidus *were inoculated intramuscularly (thigh) under isoflurane anesthesia with 100 μl of AT-2-inactivated virus (with or without 75 μg Ribi adjuvant), containing an equivalent of 5.5*10^3 ^pfu of RSV Long. FI-RSV-vaccinated animals received i.m. injection of FI-RSV diluted 1:125 in PBS, pH 7.4. Animals were boosted on day 21 after immunization with the same formulation as used for priming, and on day 42 after the initial immunization challenged with RSV Long, 3*10^5 ^pfu in 100 μl per animal. Four days after infection all animals were sacrificed by CO_2 _inhalation, lungs removed and bisected for viral titers and histopathology analyses. Live infection-immunized animals were challenged with RSV Long, 3*10^5 ^pfu in 100 μl per animal twice with an interval of 21 days and sacrificed 4 days after the second challenge. Histolopathology was analyzed as previously described [41]. In brief, lungs were intratracheally inflated with 10% neutral buffered formalin, embedded in paraffin, and sectioned at 4 μM. Sections were stained with haematoxylin and eosin (HistoServe, Gaithersburg, MD). Each lung section was scored for one of the four parameters of pulmonary inflammatory changes: peribronchiolitis (inflammatory cells, primarily lymphocytes, surrounding a bronchiole), bronchitis (neutrophils within the bronchial epithelium), alveolitis (inflammatory cells within alveolar spaces) and interstitial pneumonitis (increased thickness of alveolar walls associated with inflammatory cells [41]. Each parameter was scored individually for each section. Maximum possible value for each lesion was 4. Viral titers were determined by plaque assay [40] and expressed as geometric mean ± SE values for all cotton rats in a group. Student's t test was used to evaluate differences among groups. Histological lesion scores were also expressed as mean ± SE values for all cotton rats in a group.

### Analysis of RSV proteins by Western blotting

Virus inactivations for Western blot analysis were carried in 2 ml total volume, and otherwise were analogous to 200 μl inactivation reactions outlined above. Suspension of inactivated/control virus corresponding to 10^7.5 ^pfu/ml virion particles (as determined for control reaction, in which no inactivating agent was present) was diluted 1:2.5 with PBS, pH 7.4, after what 2× SDS-loading buffer with or without β-mercaptoethanol (20 μl/ml) was added, and samples were boiled for 5 min. Equal amounts of samples were separated on 12% Tris-Glycin Polyacrylamide Gels (Invitrogen), and were then transferred to Immobilon-P membranes (Millipore). Blots were incubated with chicken IgY antibodies derived against various RSV Long proteins. Membranes were incubated with a secondary antibody conjugated with horseradish peroxidase, and blots were developed using the ECL kit (Amersham Pharmacia Biotech).

## Competing interests

The authors declare that they have no competing interests.

## Authors' contributions

MSB conceived of the study, carried out the assays and drafted the manuscript. JCGB and GAP participated in the coordination of the study and revising the manuscript. All authors read and approved the final manuscript.

## References

[B1] Clemens R Safary A Hepburn A Roche C Stanbury WJ Andre FE Clinical experience with an inactivated hepatitis A vaccine J Infect Dis 1995 171 Suppl 1 S44 9 7876648 10.1093/infdis/171.supplement_1.s44

[B2] Murdin AD Barreto L Plotkin S Inactivated poliovirus vaccine: past and present experience Vaccine 1996 14 8 735 46 10.1016/0264-410X(95)00211-I 8817819

[B3] Murphey-Corb M Martin LN Davison-Fairburn B Montelaro RC Miller M West M Ohkawa S Baskin GB Zhang JY Putney SD Allison AC Eppstein DA A formalin-inactivated whole SIV vaccine confers protection in macaques Science 1989 246 4935 1293 7 10.1126/science.2555923 2555923

[B4] Kim HW Canchola JG Brandt CD Pyles G Chanock RM Jensen K Parrott RH Respiratory syncytial virus disease in infants despite prior administration of antigenic inactivated vaccine Am J Epidemiol 1969 89 4 422 34 4305198 10.1093/oxfordjournals.aje.a120955

[B5] Fulginiti VA Kempe CH Killed-measles-virus vaccine Lancet 1967 2 7513 468 10.1016/S0140-6736(67)90887-2 4166115

[B6] Moghaddam A Olszewska W Wang B Tregoning JS Helson R Sattentau QJ Openshaw PJ A potential molecular mechanism for hypersensitivity caused by formalin-inactivated vaccines Nat Med 2006 12 8 905 7 10.1038/nm1456 16862151

[B7] Maurer B Bannert H Darai G Flugel RM Analysis of the primary structure of the long terminal repeat and the gag and pol genes of the human spumaretrovirus J Virol 1988 62 5 1590 7 2451755 10.1128/jvi.62.5.1590-1597.1988 PMC253186

[B8] Aldovini A Young RA Mutations of RNA and protein sequences involved in human immunodeficiency virus type 1 packaging result in production of noninfectious virus J Virol 1990 64 5 1920 6 2109098 10.1128/jvi.64.5.1920-1926.1990 PMC249345

[B9] Gorelick RJ Nigida SM Jr Bess JW Jr Arthur LO Henderson LE Rein A Noninfectious human immunodeficiency virus type 1 mutants deficient in genomic RNA J Virol 1990 64 7 3207 11 2191147 10.1128/jvi.64.7.3207-3211.1990 PMC249531

[B10] Meric C Goff SP Characterization of Moloney murine leukemia virus mutants with single-amino-acid substitutions in the Cys-His box of the nucleocapsid protein J Virol 1989 63 4 1558 68 2926863 10.1128/jvi.63.4.1558-1568.1989 PMC248388

[B11] Barraud P Gaudin C Dardel F Tisne C New insights into the formation of HIV-1 reverse transcription initiation complex Biochimie 2007 89 10 1204 10 10.1016/j.biochi.2007.01.016 17383790

[B12] Wu W Henderson LE Copeland TD Gorelick RJ Bosche WJ Rein A Levin JG Human immunodeficiency virus type 1 nucleocapsid protein reduces reverse transcriptase pausing at a secondary structure near the murine leukemia virus polypurine tract J Virol 1996 70 10 7132 42 8794360 10.1128/jvi.70.10.7132-7142.1996 PMC190766

[B13] Ott DE Hewes SM Alvord WG Henderson LE Arthur LO Inhibition of Friend virus replication by a compound that reacts with the nucleocapsid zinc finger: anti-retroviral effect demonstrated in vivo Virology 1998 243 2 283 92 10.1006/viro.1998.9062 9568028

[B14] Rice WG Supko JG Malspeis L Buckheit RW Jr Clanton D Bu M Graham L Schaeffer CA Turpin JA Domagala J Gogliotti R Bader JP Halliday SM Coren L Sowder RC Arthur LO Henderson LE Inhibitors of HIV nucleocapsid protein zinc fingers as candidates for the treatment of AIDS Science 1995 270 5239 1194 7 10.1126/science.270.5239.1194 7502043

[B15] Rein A Ott DE Mirro J Arthur LO Rice W Henderson LE Inactivation of murine leukemia virus by compounds that react with the zinc finger in the viral nucleocapsid protein J Virol 1996 70 8 4966 72 8764002 10.1128/jvi.70.8.4966-4972.1996 PMC190449

[B16] Rossio JL Esser MT Suryanarayana K Schneider DK Bess JW Jr Vasquez GM Wiltrout TA Chertova E Grimes MK Sattentau Q Arthur LO Henderson LE Lifson JD Inactivation of human immunodeficiency virus type 1 infectivity with preservation of conformational and functional integrity of virion surface proteins J Virol 1998 72 10 7992 8001 9733838 10.1128/jvi.72.10.7992-8001.1998 PMC110135

[B17] Collins PL Hill MG Cristina J Grosfeld H Transcription elongation factor of respiratory syncytial virus, a nonsegmented negative-strand RNA virus Proc Natl Acad Sci USA 1996 93 1 81 5 10.1073/pnas.93.1.81 8552680 PMC40182

[B18] Hardy RW Wertz GW The product of the respiratory syncytial virus M2 gene ORF1 enhances readthrough of intergenic junctions during viral transcription J Virol 1998 72 1 520 6 9420254 10.1128/jvi.72.1.520-526.1998 PMC109403

[B19] Collins PL Hill MG Camargo E Grosfeld H Chanock RM Murphy BR Production of infectious human respiratory syncytial virus from cloned cDNA confirms an essential role for the transcription elongation factor from the 5' proximal open reading frame of the M2 mRNA in gene expression and provides a capability for vaccine development Proc Natl Acad Sci USA 1995 92 25 11563 7 10.1073/pnas.92.25.11563 8524804 PMC40442

[B20] Hardy RW Wertz GW The Cys(3)-His(1) motif of the respiratory syncytial virus M2-1 protein is essential for protein function J Virol 2000 74 13 5880 5 10.1128/JVI.74.13.5880-5885.2000 10846068 PMC112083

[B21] Berg JM Potential metal-binding domains in nucleic acid binding proteins Science 1986 232 4749 485 7 10.1126/science.2421409 2421409

[B22] Henderson LE Copeland TD Sowder RC Smythers GW Oroszlan S Primary structure of the low molecular weight nucleic acid-binding proteins of murine leukemia viruses J Biol Chem 1981 256 16 8400 6 6267042

[B23] Worthington MT Amann BT Nathans D Berg JM Metal binding properties and secondary structure of the zinc-binding domain of Nup475 Proc Natl Acad Sci USA 1996 93 24 13754 9 10.1073/pnas.93.24.13754 8943007 PMC19415

[B24] Worthington MT Pelo JW Sachedina MA Applegate JL Arseneau KO Pizarro TT RNA binding properties of the AU-rich element-binding recombinant Nup475/TIS11/tristetraprolin protein J Biol Chem 2002 277 50 48558 64 10.1074/jbc.M206505200 12324455

[B25] Amann BT Worthington MT Berg JM A Cys3His zinc-binding domain from Nup475/tristetraprolin: a novel fold with a disklike structure Biochemistry 2003 42 1 217 21 10.1021/bi026988m 12515557

[B26] Gupta CK Leszczynski J Gupta RK Siber GR Stabilization of respiratory syncytial virus (RSV) against thermal inactivation and freeze-thaw cycles for development and control of RSV vaccines and immune globulin Vaccine 1996 14 15 1417 20 10.1016/S0264-410X(96)00096-5 8994316

[B27] Dickens LE Collins PL Wertz GW Transcriptional mapping of human respiratory syncytial virus J Virol 1984 52 2 364 9 6492254 10.1128/jvi.52.2.364-369.1984 PMC254535

[B28] Rice WG Schaeffer CA Harten B Villinger F South TL Summers MF Henderson LE Bess JW Jr Arthur LO McDougal JS Orloff SL Mendeleyev J Kun E Inhibition of HIV-1 infectivity by zinc-ejecting aromatic C-nitroso compounds Nature 1993 361 6411 473 5 10.1038/361473a0 8429889

[B29] Routledge EG Willcocks MM Morgan L Samson AC Scott R Toms GL Heterogeneity of the respiratory syncytial virus 22K protein revealed by Western blotting with monoclonal antibodies J Gen Virol 1987 68 Pt 4 1209 15 10.1099/0022-1317-68-4-1209 3572360

[B30] Grosfeld H Hill MG Collins PL RNA replication by respiratory syncytial virus (RSV) is directed by the N, P, and L proteins; transcription also occurs under these conditions but requires RSV superinfection for efficient synthesis of full-length mRNA J Virol 1995 69 9 5677 86 7637014 10.1128/jvi.69.9.5677-5686.1995 PMC189426

[B31] Yu Q Hardy RW Wertz GW Functional cDNA clones of the human respiratory syncytial (RS) virus N, P, and L proteins support replication of RS virus genomic RNA analogs and define minimal trans-acting requirements for RNA replication J Virol 1995 69 4 2412 9 7884888 10.1128/jvi.69.4.2412-2419.1995 PMC188915

[B32] Fearns R Collins PL Role of the M2-1 transcription antitermination protein of respiratory syncytial virus in sequential transcription J Virol 1999 73 7 5852 64 10364337 10.1128/jvi.73.7.5852-5864.1999 PMC112646

[B33] Zamora M Samal SK Sequence analysis of M2 mRNA of bovine respiratory syncytial virus obtained from an F-M2 dicistronic mRNA suggests structural homology with that of human respiratory syncytial virus J Gen Virol 1992 73 Pt 3 737 41 10.1099/0022-1317-73-3-737 1312130

[B34] Tang RS Nguyen N Cheng X Jin H Requirement of cysteines and length of the human respiratory syncytial virus M2-1 protein for protein function and virus viability J Virol 2001 75 23 11328 35 10.1128/JVI.75.23.11328-11335.2001 11689613 PMC114718

[B35] Kim HW Arrobio JO Brandt CD Wright P Hodes D Chanock RM Parrott RH Safety and antigenicity of temperature sensitive (TS) mutant respiratory syncytial virus (RSV) in infants and children Pediatrics 1973 52 1 56 63 4353352

[B36] Crowe JE Jr Bui PT Siber GR Elkins WR Chanock RM Murphy BR Cold-passaged, temperature-sensitive mutants of human respiratory syncytial virus (RSV) are highly attenuated, immunogenic, and protective in seronegative chimpanzees, even when RSV antibodies are infused shortly before immunization Vaccine 1995 13 9 847 55 10.1016/0264-410X(94)00074-W 7483808

[B37] Wright PF Shinozaki T Fleet W Sell SH Thompson J Karzon DT Evaluation of a live, attenuated respiratory syncytial virus vaccine in infants J Pediatr 1976 88 6 931 6 10.1016/S0022-3476(76)81044-X 178852

[B38] Piedra PA Wyde PR Castleman WL Ambrose MW Jewell AM Speelman DJ Hildreth SW Enhanced pulmonary pathology associated with the use of formalin-inactivated respiratory syncytial virus vaccine in cotton rats is not a unique viral phenomenon Vaccine 1993 11 14 1415 23 10.1016/0264-410X(93)90170-3 7508665

[B39] Stevens WW Falsey AR Braciale TJ RSV 2007: recent advances in respiratory syncytial virus research Viral Immunol 2008 21 2 133 40 10.1089/vim.2008.0012 18570587 PMC3140300

[B40] Prince GA Jenson AB Horswood RL Camargo E Chanock RM The pathogenesis of respiratory syncytial virus infection in cotton rats Am J Pathol 1978 93 3 771 91 362946 PMC2018360

[B41] Prince GA Curtis SJ Yim KC Porter DD Vaccine-enhanced respiratory syncytial virus disease in cotton rats following immunization with Lot 100 or a newly prepared reference vaccine J Gen Virol 2001 82 Pt 12 2881 8 11714962 10.1099/0022-1317-82-12-2881

